# Torsion of an accessory spleen: a rare case preoperatively diagnosed and cured by single-port surgery

**DOI:** 10.1186/s40792-015-0101-x

**Published:** 2015-10-07

**Authors:** Maiko Ozeki, Mitsuhiro Asakuma, Nakai Go, Takeshi Ogura, Yoshihiro Inoue, Tetsunosuke Shimizu, Fumitoshi Hirokawa, Kazuhiro Yamamoto, Michihiro Hayashi, Yoshifumi Narumi, Kazuhide Higuchi, Kazuhisa Uchiyama

**Affiliations:** Department of General and Gastroenterological Surgery, Osaka Medical College, 2-7 Daigaku-cho, Takatsuki, Osaka 569-8686 Japan; Department of Radiology, Osaka Medical College, Takatsuki, Osaka 569-8686 Japan; Second Department of Internal Medicine, Osaka Medical College, Takatsuki, Osaka 569-8686 Japan

**Keywords:** Single-port surgery, Laparoscopic surgery, Surgical glove, Accessory spleen, Acute abdomen, Torsion, Preoperative diagnosis

## Abstract

We report a very rare case of acute abdomen caused by torsion of an accessory spleen that was preoperatively diagnosed and cured by single-port surgery. A 31-year-old woman was admitted to our hospital with severe left abdominal pain. Physical examination revealed a left upper quadrant abdominal tenderness with voluntary guarding. Ultrasound demonstrated a well-defined round mass isoechoic to the spleen, measuring 3.0 cm in diameter in the left upper quadrant adjacent to the spleen. A contrast-enhanced CT scan showed a normally enhanced spleen and a 3.0 × 3.0, hypodense, non-enhancing mass anterior to the spleen with a twisted funicular structure. Torsion of an accessory spleen was suspected, and emergency single-port surgery was performed. During surgery, a rounded violet mass measuring 3.0 cm in diameter, suggestive of an accessory spleen, with a 1800° torsion around a long vascular pedicle along the left side of the greater omentum was discovered. The mass was removed and post-operative recovery was uneventful. A review of the literature revealed 26 cases (including ours) of torsion of an accessory spleen in English. Even with the recent advances in radiologic imaging modalities, making a preoperative diagnosis of this is difficult and most cases are diagnosed during laparotomy. This is the first report preoperatively diagnosed and cured by single-port surgery. We decided to start the operation by using a single port, not only for cosmetic reasons for this young female patient, but also for final confirmation of our diagnosis. We believe that single-port laparoscopy is valuable as a diagnostic tool as long as safety is assured for patients with acute abdomen. Although torsion of an accessory spleen is extremely rare, it should be considered in the differential diagnosis of acute abdomen in children and young adults.

**Electronic supplementary material:**

The online version of this article (doi:10.1186/s40792-015-0101-x) contains supplementary material, which is available to authorized users.

## Background

Accessory spleen is a congenital anomaly characterized by ectopic tissue separated from the main body of the spleen. It is a relatively common condition that appears in 10 to 30 % of autopsy findings and is usually asymptomatic [1, 2]. It is diagnosed incidentally in radiologic examinations carried out for other reasons. However, it seldom gives rise to symptoms and very rarely involves torsion. Its clinical presentation is characterized by a non-specific acute onset or recurrent abdominal pain. Surgical removal leads to prompt recovery, but preoperative diagnosis in an emergency situation is extremely difficult, even with modern imaging techniques [2, 3].

Herein we report a rare case of an acute torsion of an accessory spleen as an emergency acute abdomen case in a young female patient, that was successfully diagnosed preoperatively. She was subsequently operated on using laparoscopic single-port surgery which has recently been developed. To the best of our knowledge, this is the first acute case of treatment by single-port surgery following preoperative diagnosis.

## Case presentation

**Fig. 1:**
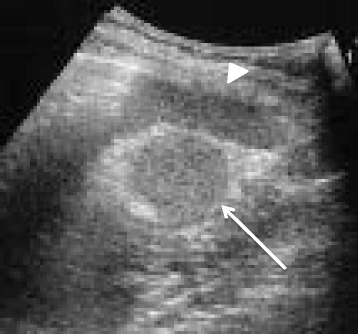
US findings. US demonstrated a round mass measuring 3.0 cm (*arrow*) which was isoechoic to the spleen (*arrow head*)

**Fig. 2:**
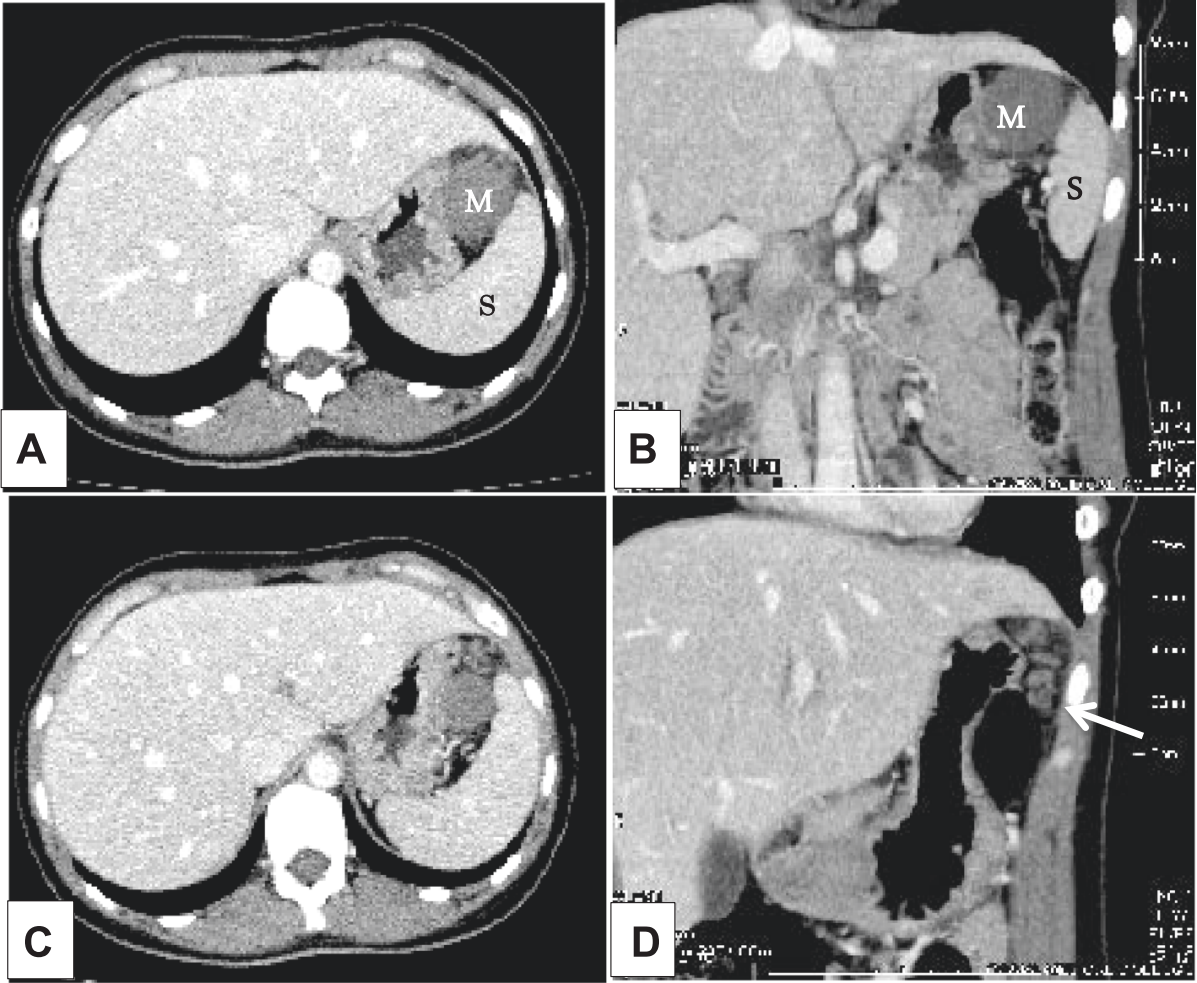
Axial and coronal contrast-enhanced CT images (**a** axial, **b** coronal). This study showed a normally enhanced spleen (S) in left upper quadrant and a non-enhancing mass (M) anterior to the spleen (**c** axial, **d** coronal). The twisted tubular structure (*arrow*) attached to the mass was detected

A 31-year-old otherwise healthy woman was admitted as an emergency with intense left abdominal pain. At the time of admission, she had pyrexia (38.5 °C). Physical examination revealed a left upper quadrant abdominal tenderness with voluntary guarding. She was only found to have an elevated level of serum C-reactive protein. Complete blood cell count was unremarkable. Ultrasound (US) demonstrated a well-defined round mass isoechoic to the spleen, measuring 3.0 cm in diameter in the left upper quadrant adjacent to the spleen (Fig. 1). Contrast-enhanced computer tomography (CT) showed a normally enhanced spleen and a 3.0 × 3.0, hypodense, non-enhancing mass anterior to the spleen with a twisted funicular structure (Fig. 2). This twisted funicular structure is more evident in a CT movie (see Additional file 1). Following diagnosis of a highly suspected torsion of an accessory spleen, we operated on her.

**Fig. 3:**
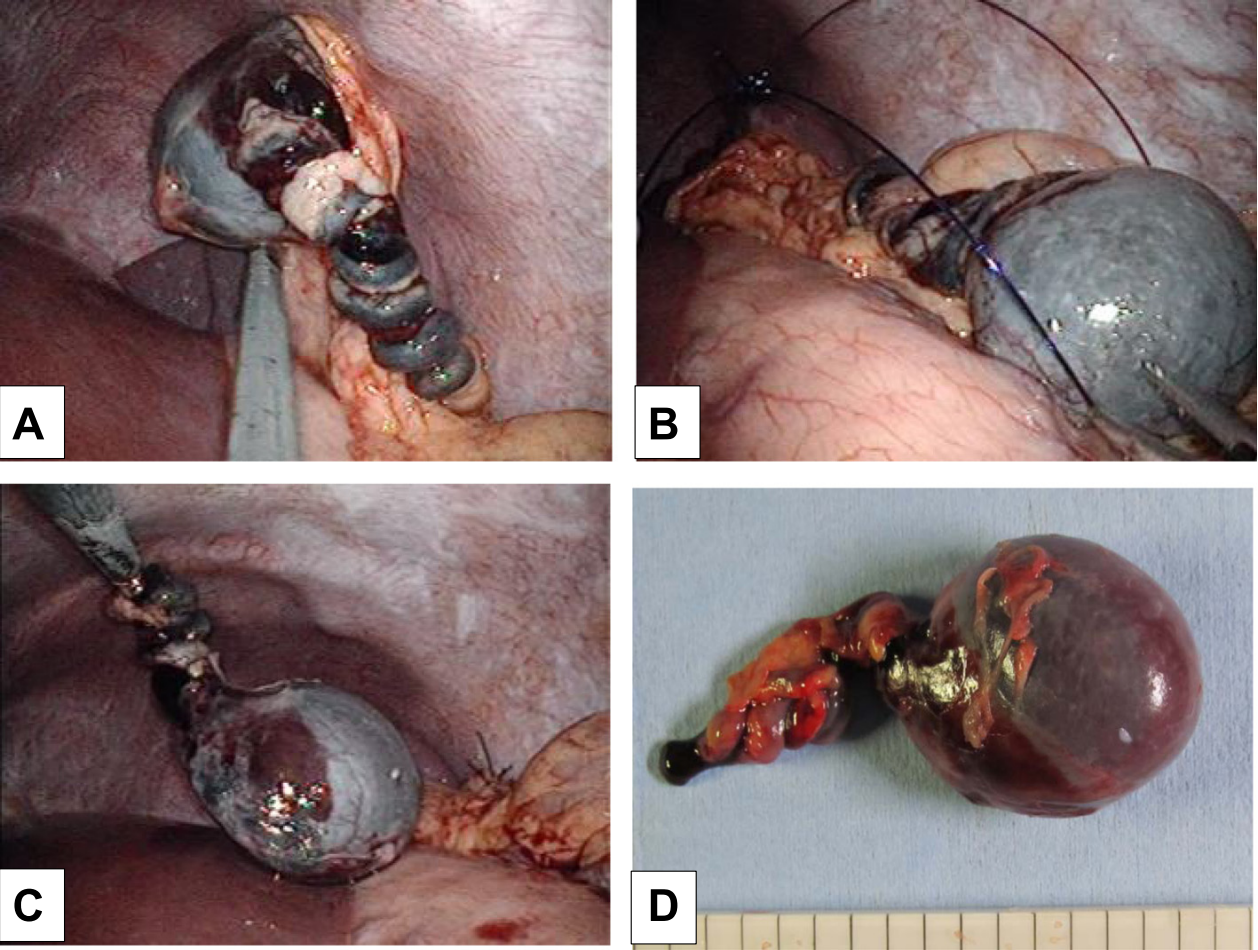
Intraoperative image highlights. **a** The rounded violet mass measuring 3.0 cm in diameter with a 1800° torsion of its long vascular pedicle. **b**, **c** The mass and its pedicle were removed using Surgitie™ loop. **d** Resected specimen showing an accessory spleen with hemorrhagic infarction and torsion of the stalk

**Fig. 4:**
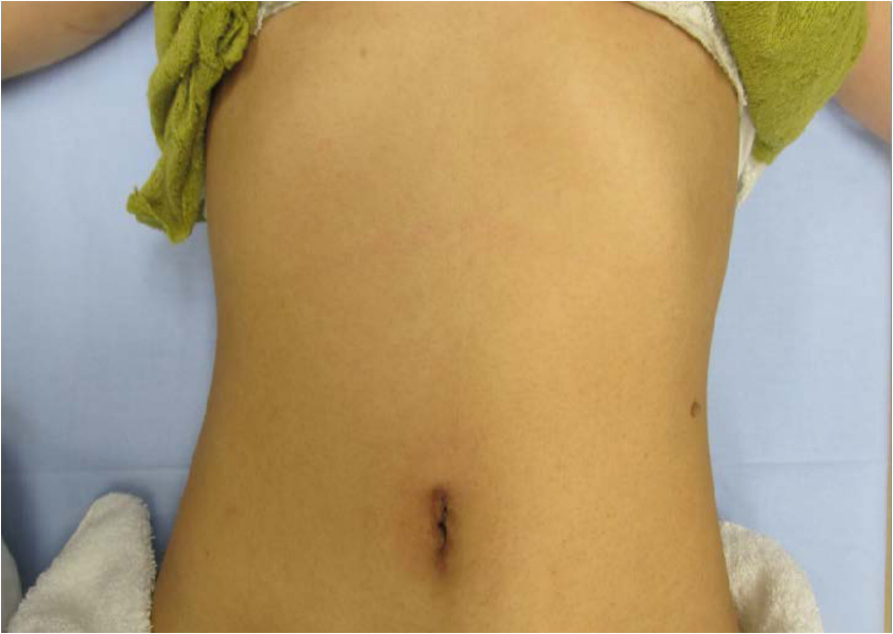
Post-operative abdomen. It shows a scarless result by single-port laparoscopic surgery

The operation was performed by single-port laparoscopy. Surgical glove method with a 1.5-cm umbilical incision was used, as we have previously reported [4, 5]. Operative findings revealed that the main spleen was normal. A rounded violet mass measuring 3.0 cm in diameter, suggestive of an accessory spleen, with a 1800° torsion around a long vascular pedicle along the left side of the greater omentum was discovered. The mass and its pedicle were removed easily, using Surgitie™ loop (Fig 3). The specimen was retrieved by Endo Catch™. Figure 4 shows the post-operative abdomen.

## Discussion

Accessory spleens are found in 10 to 30 % of autopsy findings [1, 2]. They can be solitary or multiple, are usually asymptomatic and are diagnosed incidentally in radiologic examinations carried out for other reasons. An accessory spleen may be found in various locations from the splenic hilus to the left scrotum, and is caused by the failure of the splenic anlage to fuse during embryogenesis [3, 6].

An accessory spleen without an underlying disease such as a haematological disorder or cirrhosis of the liver may occasionally present as an enlarged mass and/or acute abdomen. Emergency surgical intervention may be necessary if these events are accompanied by infarction, haemorrhage or rupture. Torsion of an accessory spleen leading to acute abdomen has been reported in literature since Alexander and Romanes first reported it in 1914 [7]. Torsion of an accessory spleen with resultant infarction may cause an acute abdomen at any age [8]. It is an extremely rare entity that is rarely diagnosed preoperatively [2, 3]. A review of the literature revealed 26 cases (including ours) of torsion of an accessory spleen after searching for the terms “accessory spleen”, “torsion” and “infarction” in PubMed [2, 3, 6–28]. Among the reported 26 cases, in 16 cases, we can find description of the size. The smallest twisted accessory spleen was 2 cm, the largest one was 17 cm and the median was 6 cm in diameter. These accessory spleens are originated in various places but mainly at the greater momentum and splenic hilus. Others were at the cecum, jejunum, mesentery and pancreatic tail. Among them, we could not find any relationship between size and tendency of torsion and origin, and also, we could not find a relationship between the length of the pedicle or the origin and torsion. Actually, only three cases were pre-diagnosed and only four cases were successfully treated by laparoscopic surgery (Table 1). This is the first report of preoperatively diagnosed torsion of an accessory spleen cured by single-port surgery. US and CT were performed in some cases. These two diagnostic tools are helpful for detecting a mass and evaluating its size, shape and influence upon surrounding tissues but are less beneficial for making a quantitative diagnosis such as neoplasm, hypertrophy, inflammation or other. Magnetic resonance imaging (MRI) has an advantage over US and CT, however, not only for detecting a mass but also for evaluating its nature and inferring its pathology, despite not always being available in an emergency situation [3, 29]. Angiography and scintigraphy were used in some cases [30]. However, making the correct diagnosis can be difficult even with angiography and scintigraphy because they are not helpful for detecting an accessory spleen when the afferent blood vessels are completely occluded.

**Table 1:** Six cases of torsion of the accessory spleen. Case reports of preoperatively diagnosed and/or cured by laparoscopic surgery in 26 cases of torsion of the accessory spleen

Author	Year	Age	Sex	Size (cm)	Location	Preoperatively diagnosed	Laparoscopic surgery
Alexander	1929	35	F	Orange	Greater omentum	○	×
Mendi	2006	12	F	NA	Splenic hilus	×	○
Yousef	2010	12	M	3.5 × 2.5 × 2	Greater omentum	×	○
Lhuaire	2013	66	M	3 × 3 × 2.5	Greater omentum	×	○
Bard	2014	20	F	17	Spleen	○	×
Our case		31	F	3 × 3 × 3.5	Greater omentum	○	Single port

*NA* not applicable

In fact, in our case, we made a successful diagnosis using both US and CT findings, which showed an isoechoic mass to the spleen and an avascular mass with twisted pedicle, respectively. In particular, the CT scan clearly showed a twisted pedicle, which was the biggest clue in making our correct diagnosis. Nonetheless, preoperative diagnosis is only hypothetical, even though it seemed correct in our case, and torsion of an accessory spleen is so rare that it is impossible to diagnose with 100 % confidence. We decided to start the operation by using a single port, not only for cosmetic reasons for this young female patient, but for final confirmation of our diagnosis. We are able to say that laparoscopy is a good diagnostic tool for acute abdomen and single-port laparoscopy is an alternative solution for diagnosing acute abdomen because if the diagnosis is different from the preoperative findings, the surgeon can easily convert to conventional laparoscopic surgery by adding as many ports as he/she needs or even switching to open surgery by making an adequate skin incision. In our case, once the diagnosis had been confirmed, we could continue with the accessory splenectomy. As an accessory splenectomy in itself is a simple procedure, the benefits for the patient are clear, i.e. much better cosmetic results and less pain than with open surgery.

## Conclusions

Torsion of an accessory spleen should be considered in the differential diagnosis of acute abdomen in children and young adults. Awareness of this entity and familiarity with typical imaging findings are important. We believe that single-port laparoscopy is valuable as a diagnostic tool, and as long as safety is assured, this method has clear benefits for patients with acute abdomen.

## Consent

Written informed consent was obtained from the patient for the publication of this case report and any accompanying images. A copy of the written consent is available for review by the Editor-in-Chief of this journal.

## Additional file

Additional file 1:
**Moving image of the CT scan.** This study shows accessory spleen and rotation of the pedicle (arrow).

## Abbreviations

CT: computed tomography
MRI: magnetic resonance imaging
US: ultrasonography
